# Integrating Vitamin A Supplementation at 6 months into the Expanded Program of Immunization in Sierra Leone

**DOI:** 10.1007/s10995-015-1706-1

**Published:** 2015-02-11

**Authors:** Mary H. Hodges, Fatmata F. Sesay, Habib I. Kamara, Emmanuel D. Nyorkor, Mariama Bah, Aminata S. Koroma, Joseph N. Kandeh, Rasmata Ouédraogo, Adam C. Wolfe, Heather I. Katcher, Jessica L. Blankenship, Shawn K. Baker

**Affiliations:** 1 Helen Keller International Sierra Leone, P.O. Box 369, Freetown, Sierra Leone; 2 Nutrition Program, Ministry of Health and Sanitation Sierra Leone, Youyi Building Brookfields, Freetown, Sierra Leone; 3 Columbia University, Mailman School of Public Health, 722 West 168th Street, New York, NY 10032 USA; 4 District Health Management Team, Western Area, Cline Town, Ministry of Health and Sanitation, Freetown, Sierra Leone; 5 West African Health Organization, Young Professional Internship Program, Helen Keller International Sierra Leone, P.O. Box 369, Freetown, Sierra Leone; 6 Helen Keller International Regional Offices, P.O. Box 13904-00800, Nairobi, Kenya; 7 Helen Keller International Regional Offices, P.O. Box BP 29.898, Dakar-Yoff, Senegal; 8 Bill and Melinda Gates Foundation, P.O. Box 23350, Seattle, WA 98102 USA

**Keywords:** Vitamin A, Expanded Program of Immunization, Infant and young child feeding, Family planning, Scaling up nutrition, Sierra Leone

## Abstract

Since 2004, twice-yearly mass vitamin A supplementation (VAS) has equitably reached over 85 % of children 6–59 months old in Sierra Leone. However infants who turn 6 months after the event may wait until they are 11 months old to receive their first dose. The effectiveness of integrating VAS at 6 months into the Expanded Program of Immunization (EPI) in a revised child health card was studied. Health facilities matched according to staff cadre and work load were assigned to provide either a ‘mini package’ of VAS and infant and young child feeding (IYCF), a ‘full package’ of VAS, IYCF and family planning (FP), or ‘child health card’ only. 400 neonates were enrolled into each group, caregivers given the new child health card and followed until they were 12 months old. More infants in the full: 74.5 % and mini: 71.7 % group received VAS between 6 and 7 months of age compared with the new CH card only group: 60.2 % (*p* = 0.002, *p* < 0.001 respectively). FP commodities were provided to 44.5 % of caregivers in the full compared with <2.5 % in the mini and new child health card only groups (*p* < 0.0001). Integration of VAS within the EPI schedule achieved >60 % coverage for infants between 6 and 7 months of age. Provision of FP and/or IYCF further improved coverage. Funding was provided by the Canadian Department of Foreign Affairs, Trade and Development who had no role in study design, data collection and analysis, decision to publish or preparation of the manuscript.

## Background

In Sierra Leone, child mortality rates are high but have declined from 214/1,000 live births in 2005 to 185/1,000 in 2011 [1]. In 2010, 22 % of children under 5 years of age were underweight, 44 % were stunted and 8 % were wasted. Infant feeding practices were sub–optimal with only 32 % of children <6 months of age being exclusively breastfed, 25 % of children 6–23 months old fed the minimal acceptable frequency and 20 % obtaining minimal dietary diversity [2]. Micronutrient malnutrition was prevalent: 76 % of children 6–59 months of age were anemic and 47 % were vitamin A deficient [3, 4].

Use of family planning commodities is low in Sierra Leone with only 8 % of women of reproductive age using modern contraception in 2011 [5, 6]. Lack of contraception use leads to minimal birth spacing and in 2011, infants born fewer than 2 years versus 3 or more years after the previous birth had more than a three-fold higher infant mortality rate: 182 versus 54/1,000 [2, 4, 7]. Globally, it is estimated that FP could prevent up to one-third of maternal deaths, while birth intervals of at least 36 months could reduce under-five mortality by 25 % [8–11].

Vitamin A supplementation (VAS) is also a key intervention to reduce child mortality, with high and sustained VAS coverage estimated to reduce mortality for children 6–59 months of age by 24 % in vitamin A deficient populations [12, 13]. Of this there is a 2.4 % reduction in infant mortality if all infants receive VAS as soon as they turn 6 months, versus the most comment current practice of twice-yearly distribution [14].

Mass VAS every 6 months through mother and child health weeks (MCHW) has been a public health success in Sierra Leone, consistently reaching over 85 % of children 6–59 months twice-yearly since 2004 [15]. However in 2012 routine VAS only reached 5 % for children between 6–11 or 12–59 months of age. The Ministry of Health and Sanitation (MoHS) is committed to universal VAS coverage as recommended by the WHO and the Global Alliance for vitamin A (GAVA), Copenhagen consensus [16, 17].

Achieving universal coverage requires reaching infants as soon as they turn 6 months, improving coverage in low-performing health districts and reaching “hard to reach” populations. As elsewhere in the region, twice-yearly events are not designed to reach infants as soon as the reach 6 months.

Since MCHWs occur every 6 months, infants who turn 6 months soon after a MCHW are left unprotected during the vulnerable second half of infancy unless reached by routine health services.

The effectiveness of different mix of services, all of which include VAS at 6 months was pilot tested after integrating VAS into the Expanded Program of Immunizations’ (EPI) schedule at 6 months of age in the Western Area (WA) of Sierra Leone. In addition, the effectiveness of including counseling for mothers/caregivers on Infant and Young Child Feeding (IYCF) and modern forms of contraception following the lactational amenorrhea period were studied. This paper presents the findings on VAS coverage, IYCF practices and FP uptake at the 6 month contact point.

## Methods

### Child Health Card Integrating VAS into the EPI Schedule

A revised child health card was developed integrating routine VAS into the EPI schedule when the infant reaches 6 months of age. The revised card also illustrated the updated WHO growth charts, best practices for maternal and child health including exclusive breast feeding, appropriate complementary feeding practices, feeding of the sick child, FP, hygiene, and malaria prevention and control (“Appendix” 1 & 2).

### Selection of Sites and Enrolment of Study Participants

Twelve peripheral health units (PHUs) stratified by staff cadre and workload were assigned to provide caregivers with a revised child health (CH) card indicating a 6 month VAS contact point in addition to: (1) ‘revised CH only’ with current services by MoHS which included routine VAS; (2) ‘mini package’ with routine VAS and IYCF counseling or (3) a ‘full package’ of routine VAS, IYCF counseling and FP counseling and provision. From April 2011, 400 infants from 0 to 3 weeks of age were enrolled in each group, weighed within 24 h of delivery and followed until they were 12 months old.

### Infant and Young Child Feeding Counseling and Demonstration

Open-ended questions on immediate breastfeeding, defined as putting the baby to the breast within an hour of delivery, were asked by health workers (HWs) at enrolment. On subsequent visits, the mother was asked about her breastfeeding practices (exclusive or not). Community health volunteers (CHVs) were given training on IYCF practices and provided counselling on complementary feeding to caregivers in the full and mini groups at the 6-month contact point. Counseling included caregiver participation in hand washing with soap, hygienic care of utensils and materials, the preparation of a nutrient rich porridge of appropriate food consistency, responsive feeding and spoon feeding from a cup. Supervision by the CHV also aimed to correct common malpractices such as forceful- and hand-feeding.

### Family Planning Counseling and Provision of Commodities

Dedicated, routine FP services were provided by a trained, certified Nursing Aid employed by Marie Stopes-Sierra Leone (MSSL) for individuals in the full package group. FP counseling was conducted in an allocated, private side-room for mothers bringing their child for their 6 month visit. Oral/injectable contraceptives and condoms were displayed, indications and possible side effects were discussed before a choice was made and commodities were then provided free of charge. A follow-up FP appointment was made for further counseling and delivery of FP commodities during the infant’s next scheduled visit for measles vaccination at 9-month of age. If a longer-term method (implants, IUDs or sterilization) were selected by the mother, a referral was made to a MSSL clinic indicating that initial counseling had been performed in this ‘outreach’ setting and a longer term method was the mother’s choice.

### Growth Monitoring

Growth monitoring by weighing and plotting on the CH chart is a regular procedures at each visit to a health unit for either EPI or illness. In all groups, weights should be measured and recorded at all visits.

### Training and Supportive Supervision

Four, one-day trainings were conducted by Helen Keller International (HKI) and the District Health Management Team (DHMT)-WA to inform HWs of the new services to be provided at the 6-month contact point. All training materials were approved by the MoHS Nutrition Department and followed national guidelines. Supportive supervision of HWs was provided by the DHMT-WA and MoHS nutrition staff.

### Monitoring and Evaluation

The study was monitored by the MoHS on a monthly basis using a standardized monitoring checklist to track progress and identify challenges to HW/CHV performance. The monitoring checklist measured input indicators including availability of child health cards, vitamin A capsules, FP and IYCF commodities, accuracy of weighing scales, and the quality of service-delivery. Weaknesses were reported immediately and corrective measures were taken by the DHMT-WA and/or HKI.

### Data Collection and Recording

All enrolled infants received a revised child health card with a unique identification code. The revised health cards were dissimilar to the current cards in size and color, making them easy to identify in the busy PHUs. Trained HKI enumerators visited on child health days and interviewed the enrolled mothers/caregivers on IYCF and FP services. They also inspected the PHU records to collect data on infants that had attended in the interim. House visits were conducted to track those lost to follow up. The data were recorded in ledgers and entered into the database.

### Statistical Analysis

The sample size of 400 per group was set assuming a lost to follow-up rate of 25 %. Data was routinely cleaned by comparing the hard copy ledgers against the Excel 2007 database and analyzed using SPSS V20 (IBM, USA). Software for emergency nutrition assessment (ENA) was used to convert infant weights to weight/age Z scores (WAZ). Low birth weight was defined as <2.5 kg measured within 24 h of delivery. Moderate and severe under-weight was defined as WAZ <2 and <3SD respectively. Statistical significance was tested by ANOVA and Chi square tests.

### Missing Data

Infants not brought to the PHUs at 12 months of age were traced by home visits whenever possible. In the event an infant was reported to have died, enumerators tried to trace and interview family members to elicit the cause of death using a simple questionnaire of signs and symptoms including cough, diarrhea and fever (including malaria). Only responses recorded as ‘yes’ for IYCF and FP at the 6 months contact point were used in the analysis.

### Ethical Considerations

The MoHS Ethics and Scientific Review Committee of Sierra Leone approved the study. As literacy rates are low, informed verbal consent was obtained from caregivers [18].

## Results

### Sample Size by Sex

A total of 1,200 infants were enrolled, with the total sample consisting of 49.3 % males and 50.7 % females. There were no significant differences in sex overall or by group.

### Routine Vas Coverage at the 6-Month Contact Point

Overall routine VAS coverage between 6 and 7 months of age was 69.4 % (95 % CI 68.9–70.3 %). Significantly higher routine VAS coverage was observed in the full: 74.5 % (95 % CI 73.7–75.3 %) and mini: 71.7 % (95 % CI 71.7–72.6 %) versus the ‘revised CH only’ group: 60.2 % (95 % CI 59.8–61.1 %) (*p* = 0.002, *p* < 0.001 respectively). Overall the average age of receipt of routine VAS was 6.2 months of age: range 6.1–6.3 months by group.

### IYCF Practices, Counseling and Demonstrations

Almost half of mothers reported having breastfed their infant within 1 h after birth, with early initiation significantly higher in both the full and mini versus the revised CH card group (*p* < 0.0001) (Table 1). Significantly higher rates of exclusive breast feeding (EBF) were recorded in the full versus revised CH card only group up to 4 months of age (*p* < 0.0001). Overall, at 6 months of age 12.4 % (95 % CI 10.5–14.3 %) of mothers were still exclusively breastfeeding.

**Table 1:** Breast feeding practices from birth to six months, IYCF and FP services at the 6 months contact point

Indicator	Overall (%)	Full (%)	Mini (%)	New CH only (%)	Significance *p* value
Immediate BF	541/1,196 (45.2)	224/400 (56.0)	213/400 (53.2)	104/396 (26.2)	Higher in both F and M versus C *p* < 0.0001
Exclusive breast feeding					
1 < 2 months	829/1,115(74.3)	290/399 (72.7)	340/400 (85.0)	199/396 (50.2)	Higher in both F and M versus C *p* < 0.0001 and M versus F *p* = 0.00101
2 < 3 months	276/510 (54.1)	115/143 (80.4)	104/216 (48.1)	57/151 (37.7)	Higher in F versus both M and C *p* < 0.0001
3 < 4 months	161/475 (33.9)	81/137 (59.1)	55/213 (25.8)	25/125 (20)	Higher in F versus M *p* < 0.0005 and C *p* < 0.0001
4 < 5 months	94/440 (21.4)	50/124 (40.3)	29/212 (13.7)	15/104 (14.4)	Higher in F versus M *p* = 0.007 and F versus C *p* = 0.021
5 < 6 months	90/728 (12.4)	56/240 (23.3)	15/342 (4.4)	19/146 (13)	Higher in F versus M *p* = 0.016
IYCF					
IYCF counseling	518/1,155 (44.8)	237/380 (62.4)	270/378 (71.4)	11/397 (2.8)	Higher in both F and M versus C *p* < 0.0001
CF preparation	511/1,155 (44.2)	238/380 (62.6)	271/378 (71.7)	2/397 (0.5)	Higher in both F and M versus C *p* < 0.0001
FP services					
FP counseling	259/1,155 (22.4)	235/380 (61.8)	18/378 (4.8)	6/397 (1.5)	Higher in F versus both M and C *p* < 0.0001
FP commodities	181/1,155 (15.7)	169/380 (44.5)	9/378 (2.4)	3/397 (0.8)	Higher in F versus both M and C *p* < 0.0001

Counseling on IYCF and caregiver participation in the preparation of a nutrient rich porridge was provided to over 62.4 % (95 % CI 61.4–63.5 %) of caregivers in the full and over 71.4 % (95 % CI 70.1–72.7 % in the mini group compared to 2.8 and 0.5 % (95 % CI 0.0–1.77 %) in the revised CH card only group respectively (*p* < 0.0001 both).

### Family Planning Services at the 6-Month Contact Point

Counseling for FP was provided to 61.8 % (95 % CI 60.8–62.9 %) of caregivers in the full compared with 4.8 % (95 % CI 2.9–6.8 %) in the mini and 1.5 % (95 % CI 0.0–3.2 %) in the revised CH card only group (*p* < 0.0001 for both) (Table 1). Commodities were provided to 44.5 % (95 % CI 43.2–45.9 %) of caregivers in the full compared with 2.4 % (95 %CI 0.8-5.4 %) in the mini: and 0.8 % (95 % CI 0.0–2.6 %) in and revised CH only group (*p* < 0.0001 both). Overall 69.9 % of women counselled on FP at the 6 months contact point accepted FP commodities (181/259).

### Vaccination Status at the 6-Month Contact Point

There was no significant difference in the number of fully vaccinated infants between groups, full package: 95.8 % (95 % CI 95.1–96.2 %), mini package: 96.6 % (95 % CI 96.3–97.1 %), and revised CH card only: 92.4 % (95 % CI 92.2–93.0 %). However significantly more infants received ‘catch-up’ vaccinations (doses 2 or 3) of polio, pentavalent and/or PCV in both the full and mini package versus the revised CH card only (*p* = 0.019, *p* = 0.003 respectively). In total 6.1 % (CI 5.5–6.5 %) infants received a ‘catch-up’ vaccination at the 6 month contact point.

### Infant Growth

At enrolment there were no significant differences between groups in mean WAZ (Table 2).

**Table 2:** Mean WAZ (±2SD) and percentage underweight by intervention and age group

Age	Overall				Full				Mini				New CH only			
	N	WAZ	<− 2 SD WAZ	% underweight	N	WAZ	<− 2 SD WAZ	% underweight	N	WAZ	<− 2 SD WAZ	% underweight	N	WAZ	<− 2 SD WAZ	% underweight
≤21 days	1,179	0.14 ± 1.55	71	6.0	389	0.43 ± 1.51	12	3.1	396	0.04 ± 1.51	30	7.6	394	0.05 ± 1.58	29	7.4
6 < 9 months	935	−0.99 ± 1.35	195	20.8	253	−0.56 ± 1.14	29	11.5	308	−0.78 ± 1.16	43	14.0	374	−1.45 ± 1.48	123	32.9
9 < 12 months	918	−0.02 ± 1.12	53	5.8	238	0.24 ± 0.98	5	2.1	330	−0.05 ± 1.05	15	4.5	350	−0.15 ± 1.24	33	9.4

There was no significant different by group at enrolment or between full versus mini at 6 < 9 months of age. There was a significantly higher WAZ in the full versus mini at 9 < 12 (*p* = 0.013), full versus new CH card only at 6 < 9 and 9 < 12 (*p* < 0.0001 both), mini versus new CH card only at 6 < 9 and 9 < 12 (*p* < 0.0001, *p* = 0.065)

The overall prevalence of low birth weight was 2.8 % (95 % CI 2.2–3.5 %) with no significant difference between groups full: 1.5 % (95 % CI 1.0–2.1 %), mini: 3.0 % (95 % CI 2.4–3.8 %) and revised CH card only: 3.8 % (95 % CI 3.2–4.5 %).

At 6 < 9 months of age, the mean WAZ was significantly higher for both the full and mini versus the revised CH only group (*p* < 0.0001). The overall prevalence of moderate underweight was 12.6 % (95 % CI 5.7–25.5 %) and severe underweight was 8.2 % (95 % CI 0.6–58.8 %).

At 9 < 12 months of age, the mean WAZ was significantly higher for the full versus mini and full versus revised CH only groups (*p* < 0.005, *p* < 0.0001 respectively) and a trend towards significance in the mini versus revised CH card only group (*p* = 0.065). The overall prevalence of moderate underweight was 4.8 % (95 % CI 1.0–19.8 %) and severe underweight was 1.0 % (95 % CI 0.1–7.2 %).

### Loss to Follow Up

**Fig. 1:**
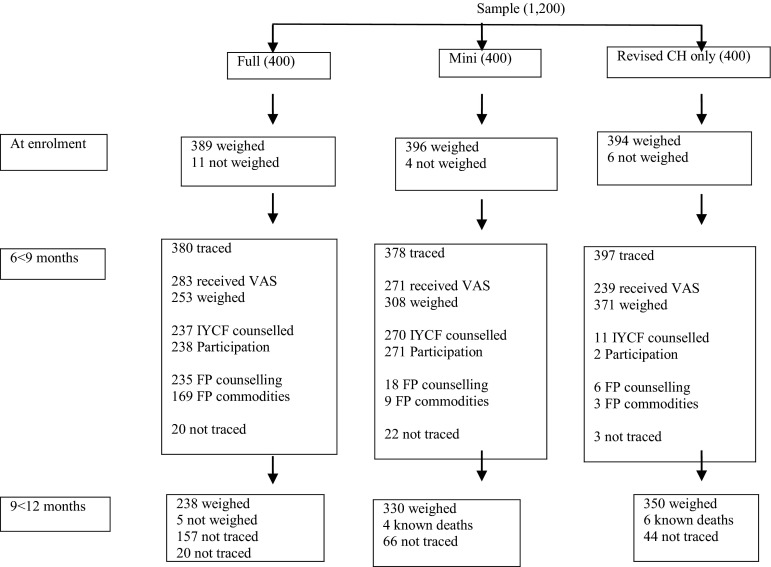
Consort diagram of missing data

Overall, WAZ were obtained for 77.9 % (935) and 76.5 % (918) of infants at 6 < 9 and 9 < 12 months of age, respectively (Fig. 1). At 9 < 12 months of age of those lost to follow up, 202 were reported to have moved out of the area, 52 names and/or addresses were un-traceable by house visits and 10 infants were known to have died.

## Discussion

Routine coverage of VAS at 6–7 months of age increased to over 60 % in all groups with the introduction of the revised CH card from 5 % for infants aged 6–11 months of age. This increase uptake of VAS could in part be due to other factors such as better recording, reporting or supportive supervision rather than the child health card. However, this modification to the EPI schedule, if implemented at scale, could complement twice-yearly events to ensure universal VAS coverage, helping to reach ‘hard to reach’ infants and reducing malnutrition and mortality [19, 20].

The significantly higher rates of immediate breast feeding and EBF to 2 months observed in the full and mini groups versus the revised CH card only group make assessing the impact of IYCF services at the 6 month contact point difficult. It is possible, that the IYCF counseling and participation contributed to the differences between groups via passing of information by word of mouth from caregivers with older infants in the full and mini groups.

The turn-over of CHVs performing the IYCF counseling and demonstration was high, but the training/refresher training in IYCF could have also contributed to the results. CHVs are frequently either young school-leavers awaiting acceptance into a health-training institution or more senior community members with limited education. Progress is being made by the MoHS and partners in the recognition of the important role CHVs play in maintaining a wide variety of health services and a small stipend/allowance is becoming increasingly practiced in order to retain quality services. Assigning this role to local, recognized Traditional Birth Attendants could help re-enforce the links between communities.

Although IYCF and FP counseling and provision of free commodities is part of the MoHS’ essential health basic package at the PHU level these services are often not provided as demonstrated by the revised CH card only findings [21]. Issues with provision of FP counseling and provision include inadequate number of trained HWs, lack of supplies, low prioritization given to re-ordering of FP supplies and low motivation to give FP commodities free of charge. Overall a remarkably high proportion of women requested and were supplied FP commodities when private counseling was offered as routine at the 6 months contact point, demonstrating the current contraceptive gap [22].

The loss to follow up by subjects moving out of the area is not unusual in post-conflict Sierra Leone. Rapid population migration has been observed by other programs with rural–urban–rural transitioning depending upon employment-seeking, farming commitments and school holidays [23].

Based on the results of this study, the MoHS has made a commitment to nation-wide institutionalization of the integrated 6-month contact point in routine child health services as part of its ‘Agenda for Prosperity’ and as a key intervention in the Scaling up Nutrition initiative. Pre- and in- service training curricula have been developed, and training of trainers commenced in mid-2013 [24–26]. The nation-wide implementation of integrated services will require extensive training for all HWs and CHVs to support programming. Funding for has been secured and the scale-up commenced in late 2013 and were due to be completed by late 2014.

The challenges anticipated included accurate plotting and interpretation of weights for age on the revised growth charts, supply chain management of increased demand for FP commodities and lack of HWs who are currently suitably qualified to counsel mothers on the full range of FP methods. Another pilot study is being undertaken to revise the qualification requirements needed by HWs to undertake this role. The unanticipated challenge of the Ebola outbreak necessitated the cessation of training in mid-2014 and this is unlikely to be re-started until mid-2015.

### Study Limitations

A limitation of this study is that participants were recruited at the health center, which may not represent the general population. Data collectors were aware of the clinic to which interventions were assigned which may have introduced some observer bias even though they were re-assigned periodically. In addition the IYCF counselling skills of CHVs may have varied during the study period.

## Conclusions

Integrating routine VAS into the EPI schedule with revised child health cards increased routine VAS coverage at 6 months to over 60 %. Integrating IYCF and FP at the 6-month contact point further increased routine VAS coverage to over 70 %. There was a high uptake of FP services when offered as routine services.

## Figures and Tables

**Fig. 2:**
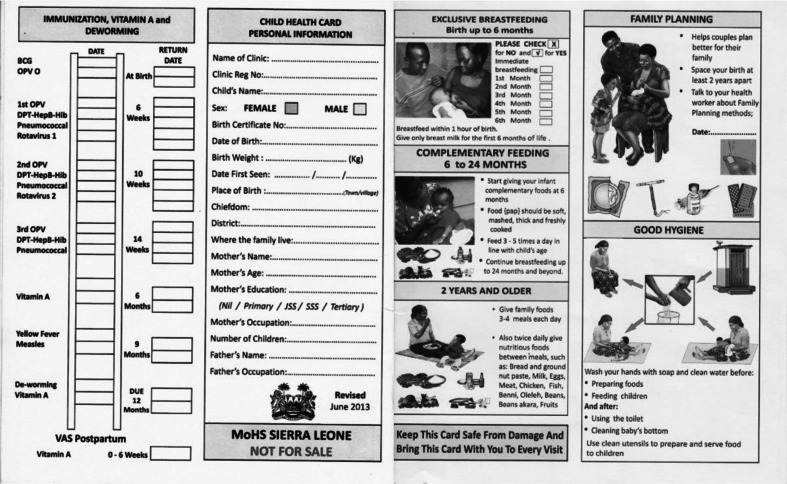
Front of 4-fold revised child health card

**Fig. 3:**
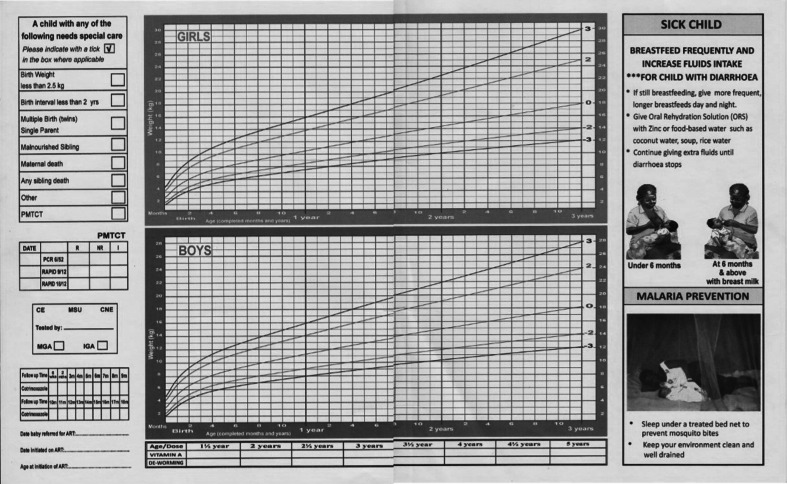
Back of 4-fold revised child health card

## Appendix

See Figs. 2 and 3.
